# Proteus syndrome with late diagnosis confirmation: a case report

**DOI:** 10.3389/fped.2025.1568806

**Published:** 2025-05-08

**Authors:** Ilona Dockienė, Martynas Čiuplinskas, Rimantas Zagorskis, Eglė Kontrimavičiūtė

**Affiliations:** Institute of Clinical Medicine, Faculty of Medicine, Vilnius University, Vilnius, Lithuania

**Keywords:** Proteus syndrome, whole exome sequencing, AKT1, tissue overgrowth, vascular malformation, difficult airway management

## Abstract

Proteus syndrome (PS) is a rare, highly polymorphous and complex disorder characterized by assymetric and/or disproportional overgrowth of limbs, hamartomas and vascular deformations. Typically, the first signs of PS appear between 6 and 18 months of age, are subtle and might be overlooked. We report a case of a 17 year old boy which was first diagnozed with PS after left limb amputation in 2021 (age of 14), though retrospectively the first signs of left hand enlargement appeared at 18 months. At the age of 3 years vascular surgery of venous malformation on his left leg was performed, molecular genetic tests from blood revealed no abnormalities. At the time Klipper-Trenaunay syndrome was diagnosed according to clinical criteria. In 2021 whole exome sequencing (WES) confirmed the diagnosis of PS after the test of affected tissues after amputation. We review the role of multidisciplinary approach implicating different physicians, role of radiologist with the multiple findings in this rare pathology with high variability of clinical presentation. Considering the complications and early mortality (up to 27 years old) observed in patients we emphasizet he significance of early suspicion and diagnosis of PS and the need for symptomatic multidisciplinary team follow-up which aims to minimize the degree of disability, prophylaxis of thrombembolic events and improve quality of life.

## 1 Introduction

Proteus syndrome (PS) (OMIM ID 176920) is a group of uncommon diseases and leads to progressive segmental tissue overgrowth, most individuals show minimal or no signs at birth (1). Typically, the first manifestations appear between 6 and 18 months of age as enlargement of feet or hands but it can occur in any part of the body. The incidence of PS is between 1:1000000 and 1:10000000. Currently, there are approximately 200 cases described in the literature (2).

PS results from a mutation in the AKT1 gene. A somatic mosaic mutation in the AKT1 oncogene was found in >90% of individuals who met the diagnostic criteria of PS (Table 1). This change is not inherited from the parents, but arises randomly in one or a group of cells during the early stages of foetal development. A combination of cells with and without mutations is known as mosaicism (3). This syndrome is lethal if mutations are carried in a nonmosaic manner. Molecular diagnosis requires sensitive methods and the analysis should be performed from affected tissues.

**Table 1:** Diagnostic criteria of Proteus syndrome (4).

General criteria	Specific criteria
1. Mosaic distribution 2. Progressive course 3. Sporadic Occurrence	Category A 1. Cerebriform connective tissue nevus
	Category B 1. Linear epidermal nevus 2. Asymmetric, disproportionate overgrowth of 2 of the following: 2.1. Limbs, skull, external auditory canal, vertebrae, or viscera 3. Specific tumors in the first decade of life 3.1. Bilateral ovarian cystadenomas 3.2. Monomorphic parotid adenomas
	Category C 1. Dysregulated adipose tissue 2. Vascular malformations of ≥1: 2.1. Capillary, venous, and/or lymphatic 3. Lung bullae 4. Facial phenotype: 4.1. Long face, dolichocephaly, downslanted palpebral fissure, low nasal bridge 4.2. Wide or anteverted nares 4.3. Open mouth at rest

Diagnosis requires all 3 general criteria plus 1 criterion from category A, 2 from category B, or 3 from category C.

The multidisciplinary management of this rare pathology involves a lot of specialists: rare disease specialist, clinical geneticists, radiologists, orthopedic surgeons, anesthesiologists, vascular surgeons, cardiologists, psychologists and many more according to prevailing health issues. The differential diagnoses included neurofibromatosis, Bannayan–Zonana syndrome, Beckwith–Wiedemann syndrome, Klipper–Trenaunay syndrome, and Maffucci syndrome.

Prognosis depends on the location, degree of tissue overgrowth and presence of serious complications. The diagnosis, follow-up, detection of complications and early prophylactic antithrombotic treatment may improve quality of life and prolong the life expectancy which is up to 27 years.

## 2 Case report

The first case of PS in Lithuania was diagnosed in 2021 in a 14-year-old boy. He was born without any obvious signs of genetic diseases, with a few haemangiomas on the back, left leg, and left buttocks. In early childhood, he was diagnosed with an atrial septal defect of the heart, which was monitored by cardiologists and eventually closed by itself at preschool age.

**Figure 1:**
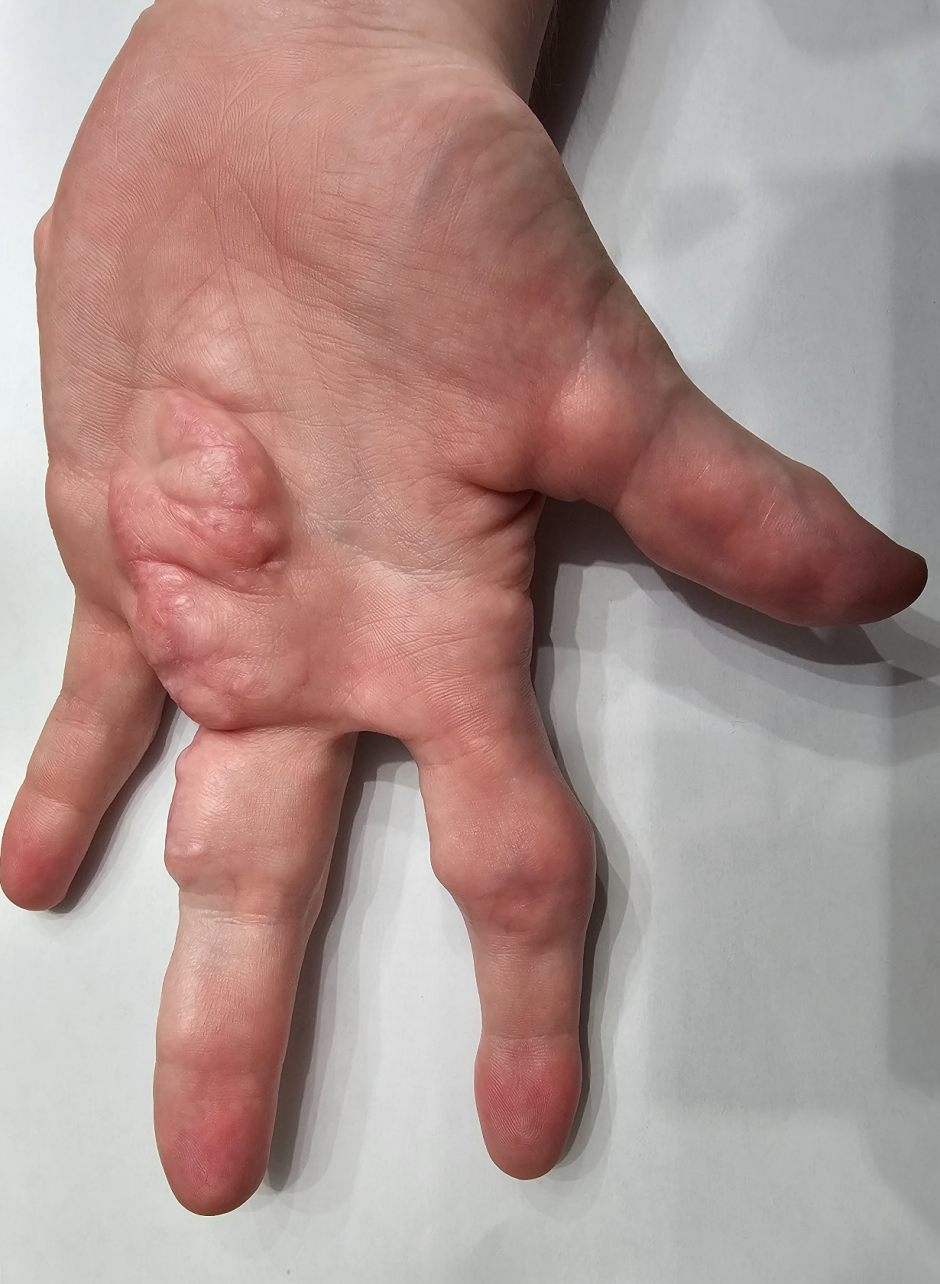
Enlargement of the fingers of the left arm.

At 18 months of age, the fingers on the left arm became enlarged (provided image shows the current view) (Figure 1). At 24 months of age, a large lipoma was observed on the left side of the patient's waist. Multiple lipomas in the armpits and testicles were found, the difference in the size of the feet became apparent, and he developed difficulties wearing shoes.

The patient was followed by vascular surgeons, negative genetic tests from blood sample were obtained at 3 years of age and Klipper–Trenaunay syndrome was diagnosed based on the clinical appearance. The patient underwent surgery on the left leg because of varicose veins, wore compressive socks postoperatively, and started antiplatelet therapy.

**Figure 2:**
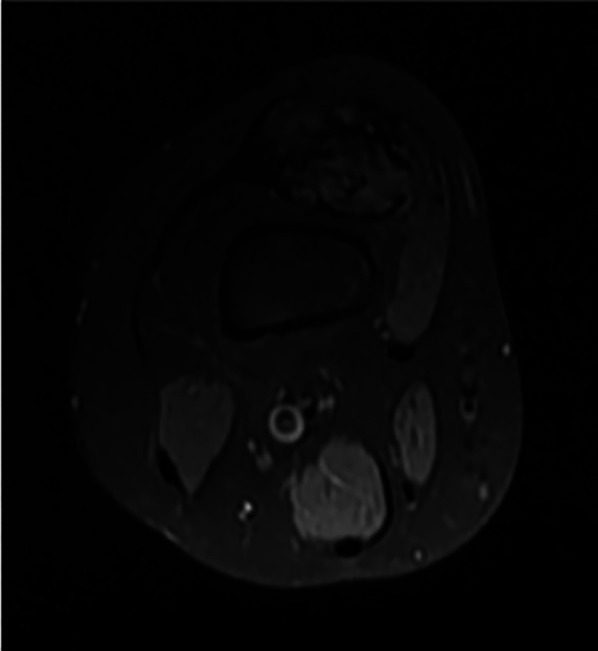
Ax T1 FS knee MRI - typical hamartoma.

**Figure 3:**
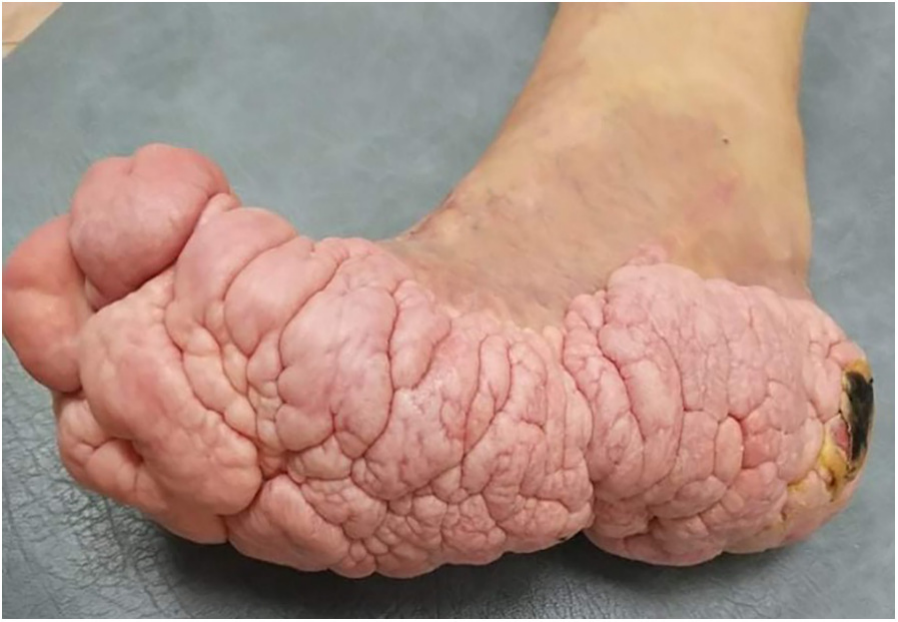
Brain-like nevuses of the left foot and non-healing infected ulcer of the altered heel.

The patient's growth spurt occurred at 11–14 years, and the disease progressed rapidly. The patient became tall and heavy for his age and developed facial features characteristic of PS. The left leg enlarged dramatically (Figure 2), and all changes in the trunk and left hand progressed further. The biggest problems started as footwear caused skin ulcers on the altered heel of the left leg and the development of nonhealing-infected ulcers (Figure 3). In 2021, amputation was performed below the knee due to the progression of vein thrombosis and non-healing ulcers. The postoperative period was complicated by impaired wound healing, requiring further surgical intervention. A leg prosthesis was fitted. Initially, the first molecular genetic testing was performed on a blood sample, but the testing must be performed on altered tissue. In 2021, biopsy of the lesions was performed, and WES test confirmed the mutation of AKT1 (genotype NM_001014431.2:c.[49=/G > A]; [49=], NP_001014431.1:[(Glu17=/Lys)];[(Glu17=)] and diagnosis of PS. The patient underwent 10 general anaesthesia procedures, some in combination with regional anaesthesia.

Currently, the 17-year-old patient is characterised by low intelligence. His abilities correspond to those of a 9-year-old, and he is studying according to a customised program. His therapeutic condition was relatively good, and he was receiving only antiplatelet therapy. The manifestation of PS slowed as the patient reached skeletal maturity. Tibial osteotomy is planned to be performed within a few months.

## 3 Discussion

PS is caused by a mosaic variant of AKT1, which activates the PI3K-AKT pathway (5). Somatic activating mutations in the PI3K/AKT/mTOR pathway are among the most common mutations identified in cancer and have been shown to cause a spectrum of overgrowth syndromes including PIK3CA-Related Overgrowth Spectrum, Proteus syndrome, and brain overgrowth conditions (6). Inclusion of PS in this group of the diseases may further contribute to research of ethiopathogenetic treatment options.

The clinical manifestations of PS are diverse, and the imaging findings are complex and lack specificity, which may lead to clinical misdiagnosis or late diagnosis. The onset of PS typically occurs in childhood, with more complex manifestations developing over time. In our case, the patient was diagnosed with Klippel–Trenaunay syndrome at 3 years of age based on clinical criteria; In 2021, WES test from affected tissue revealed a mutation in the AKT1 gene. Diagnosis was confirmed according to the diagnostic criteria for PS: general criteria (mosaic distribution, progressive course, and sporadic occurrence), two specific criteria for category B (asymmetric disproportionate overgrowth of two limbs and specific tumours in the first decade of life), and three specific criteria for category C (dysregulated adipose tissue, >1 vascular malformation, and facial phenotype).

In the report of Min He and Weijia Zhao (2020), a rare case of PS was presented in woman with massive excrescence and cerebriform plantar hyperplasia of the left foot (7). Furthermore, given that PS overgrowth typically manifests between the ages of 6 and 18 months, this case is unusual owing to the solitary cutaneous finding and delayed presentation in the fourth decade of life. The patient was diagnosed with PS based on clinical features and results of the histopathological and radiographic findings still not confirmed by WES.

On the other hand, in 2024 published case report of 34 year old woman at 20 + 2 weeks of gestation because foetal ultrasound visualised cerebral and genital anomalies with suspicion of PS (8). In this case, the radiological findings were not enough to confirm diagnosis of PS without molecular diagnosis of AKT1 gene mutation. At 21 weeks of gestation genetic analysis was performed on an amniotic fluid sample with no results, but WES identified a *de novo* pathogenic c.49G > A (p.Glu17Lys) variation in AKT1 with an allele frequency of 25%. This new information highlights the evolving role of WAS in detecting foetal mosaic pathologies.

Due to the broad range clinical features, patients with PS need to be treated by a multidisciplinary team of specialists. Specialists of rare diseases, radiologists and clinical genetics specialist play a key role in confirming the diagnosis. The radiologists interfere at so many levels of managing this rare disease, using practically different imaging tools in accordance with the highly variable manifestations of this disorder. The diagnosis, the follow-up, the detection of complications, and even the prenatal screening depend heavily on imaging findings. Moreover, psychological counseling is of great importance because many patients and their families struggle with consequences of the genetic disease. Oral and craniomaxillofacial surgeons may perform debulking of cranial overgrowths (9).

Vascular malformations are common in patients with PS. This increases the risk of deep vein thrombosis and pulmonary artery embolism even in early childhood, risk of perioperative bleeding, and need for hemotransfusions. Patients may require perioperative prophylaxis with low molecular mass heparin (10). In our case, the patient underwent surgery for varicose veins, wore compressive socks postoperatively, and started antiplatelet therapy.

Although orthopedic manifestations of PS are not the main issue of the disease, it should be considered in the treatment. Affected limb deformities, scoliosis, and soft tissue lesions are often associated with poor quality of life. Orthopaedic surgeons are able with early interventions to correct physical defects, prevent extra malformations and reduce the risk of loss of movement. Skeletal abnormalities, enlarged hamartomas, and impaired mobility caused difficulties in positioning the patient perioperatively. Our patient underwent several orthopaedic surgeries on the left hand and leg to improve quality of life.

PS may be associated with hypertrophic cardiac rhabdomyomas and conduction disorders. For patients with cardiac disease symptoms, extended cardiological examination is necessary. The minor congenital heart defect in our patient was followed by cardiologists and eventually closed.

PS is characterized by facial phenotypes of macrocephaly, abnormal teeth, macroglossia (11), overgrowth of soft tissues, asymmetric tonsils, and adenoids (12). This can lead to difficult airway management during anaesthesia. Regional anaesthesia is advantageous for orthopaedic and reconstructive surgeries because it avoids the risk of difficult intubation. Regional anaesthesia provides adequate analgesia, enables earlier mobilisation of the patient, and reduces the risk of deep vein thrombosis (13). Our patient underwent 10 uneventful general anaesthesia procedures with some difficulties in performing regional anaesthesia.

At present PS still lacks effective treatment. The current treatments are only symptomatic. Given the increasing operative difficulty and rate of complications over time, early surgical interventions are vital to reduce extra malformations, physical defects or loss of movement. However, the identification of the AKT1 mutation provides new information and theoretical basis for the diagnosis and treatment of the disease, as well as a new path for the drug development. Miransertib, which has previously been evaluated only in adult oncology trials, is an inhibitor that potently inhibits AKT 1, 2 and 3 (14). Miransertib is an allosteric inhibitor of AKT1 that has been shown *in vitro* to regulate the growth of tumors that have this variant (15). Sirolimus (rapamycin), which is used to prevent organ transplant rejection and in treatment of lymphangioleiomyomatosis and perivascular epithelioid cell tumours, is another medication that shows promise in treatment of PS (16). While the research of experimental treatment certainly provides hope for the future of treatment of PS, at the moment it is mainly focused on minimalisation of disability, increasing the quality of life and comfort without the hope for full recovery.

Prognosis is closely related to the severity of complications. Approximately 20% of patients with PS expire prematurely, commonly due to venous thromboembolism or pulmonary embolism, pneumonia, or surgical complications.

The patients ought to be periodically followed up for the development of complications. In addition, it is necessary to monitor the patients for potential tumor development.

## 4 Conclusion

Early suspicion and confirmation of PS diagnosis can be difficult and challenging due to high variability of clinical presentation. Multidisciplinary approach is paramount in follow-up throughout the patient's life considering the complications and early mortality observed.

At present PS still lacks effective treatment and current symptomatic treatments mainly aim to minimize the degree of disability, improve the quality of life and symptomatic treatment continuous thorough the patient's life. The identification of the AKT1 mutation provides new information and theoretical basis for a new path for the drug development and specific treatment for PS in the future. Individual case reports continue to help understand the disease and seek for appropriate treatment.

## Data Availability

The original contributions presented in the study are included in the article/Supplementary Material, further inquiries can be directed to the corresponding author.
